# Sex Differences in Serum Markers of Major Depressive Disorder in the Netherlands Study of Depression and Anxiety (NESDA)

**DOI:** 10.1371/journal.pone.0156624

**Published:** 2016-05-27

**Authors:** Jordan M. Ramsey, Jason D. Cooper, Mariska Bot, Paul C. Guest, Femke Lamers, Cynthia S. Weickert, Brenda W. J. H. Penninx, Sabine Bahn

**Affiliations:** 1 Department of Chemical Engineering and Biotechnology, University of Cambridge, Cambridge, United Kingdom; 2 Department of Psychiatry, VU University Medical Centre and Neuroscience Campus Amsterdam, Amsterdam, The Netherlands; 3 Neuroscience Research Australia, Schizophrenia Research Institute and University of New South Wales, Sydney, NSW, Australia; 4 Department of Neuroscience, Erasmus University Medical Centre, Rotterdam, The Netherlands; University of Oxford, UNITED KINGDOM

## Abstract

Women have a consistently higher prevalence of major depressive disorder (MDD) than men. Hypotheses implicating hypothalamic-pituitary -adrenal, -gonadal, and -thyroid axes, immune response, genetic factors, and neurotransmitters have emerged to explain this difference. However, more evidence for these hypotheses is needed and new explanations must be explored. Here, we investigated sex differences in MDD markers using multiplex immunoassay measurements of 171 serum molecules in individuals enrolled in the Netherlands Study of Depression and Anxiety (N_MDD_ = 231; N_control_ = 365). We found 28 sex-dependent markers of MDD, as quantified by a significant interaction between sex and log_2_-transformed analyte concentration in a logistic regression with diagnosis (MDD/control) as the outcome variable (*p<*0.05; *q<*0.30). Among these were a number of male-specific associations between MDD and elevated levels of proteins involved in immune response, including C-reactive protein, trefoil factor 3, cystatin-C, fetuin-A, β2-microglobulin, CD5L, FASLG receptor, and tumor necrosis factor receptor 2. Furthermore, only male MDD could be classified with an accuracy greater than chance using the measured serum analytes (area under the ROC curve = 0.63). These findings may have consequences for the generalization of inflammatory hypotheses of depression to males and females and have important implications for the development of diagnostic biomarker tests for MDD. More studies are needed to validate these results, investigate a broader range of biological pathways, and integrate this data with brain imaging, genetic, and other relevant data.

## Introduction

Major depressive disorder (MDD) is a highly prevalent and disabling condition with inadequate diagnosis and therapy [1]. In a recent WHO World Mental Health survey, the average lifetime prevalence of MDD was found to be 14.6% in high-income countries and 11.1% in middle- and low-income countries, with an approximately two-fold higher prevalence in women compared to men [2]. A higher female prevalence of MDD has been observed consistently across several countries and cultural settings from late adolescence onwards [3,4].

Several hypotheses implicate biological factors in the increased risk of MDD in females. Sex hormones have been linked to the emergence of higher rates of female depression during puberty and rising hormone levels during this time have been linked to affective disturbances in girls [5,6]. Some evidence suggests that depressed females may show greater hypothalamic-pituitary-adrenal (HPA) axis dysregulation than depressed males [7,8], while other evidence suggests the reverse, with higher baseline levels of salivary cortisol found only in medication-free depressed males [9] and in adolescent males who later developed clinical depression [10]. Extensive sex differences in immune response have also been documented [11] that may lead to sex-dependent MDD pathophysiology [12]. Subclinical hypothyroidism has been proposed to cause more MDD in females and is associated with reduced central serotonergic activity [5]. Other explanations may involve genetic factors and aspects of brain development and function [13,14]. No consistent reason for the higher rate of MDD in females has been found.

Sex-dependent peripheral changes in biological processes in MDD have implications for the development of biomarker tests. Biomarker tests to objectively diagnose MDD as part of a screening programme, to identify individuals at high risk of developing the disorder [15], and to help improve recognition and treatment of MDD in primary care settings [1] have been proposed. Changes in peripheral inflammation, oxidative stress, metabolic markers, growth factors, and endocrine factors in MDD patients are potential biomarkers [16–19] for which prominent sex differences have been found [11,20]. Sex differences in markers of MDD were reported in the work of Domenici et al. (2010) [17], who found 11 plasma analytes with significant interactions between sex and diagnosis, including growth hormone and proteins involved in immune response. However, these were not evaluated as predictive markers.

We performed an extensive investigation of sex-dependent biomarkers of MDD in order to help elucidate sex differences in the pathophysiology of MDD and explore their potential use in diagnosis. This was done using serum molecular data from the baseline assessments of 1,243 individuals enrolled in the Netherlands Study of Depression and Anxiety (NESDA), a longitudinal, multi-site naturalistic cohort study [21]. We analyzed 171 serum molecules measured using a pre-selected multiplex immunoassay panel comprised of cytokines, hormones, growth factors, metabolic markers, acute phase reactants, central nervous system markers, and others, many of which have been linked to mental disorders [19,22–24]. These analyses will benefit the development of diagnostic biomarker tests and further the understanding of molecular mechanisms underlying the disorder in males and females.

## Methods

**Fig 1:**
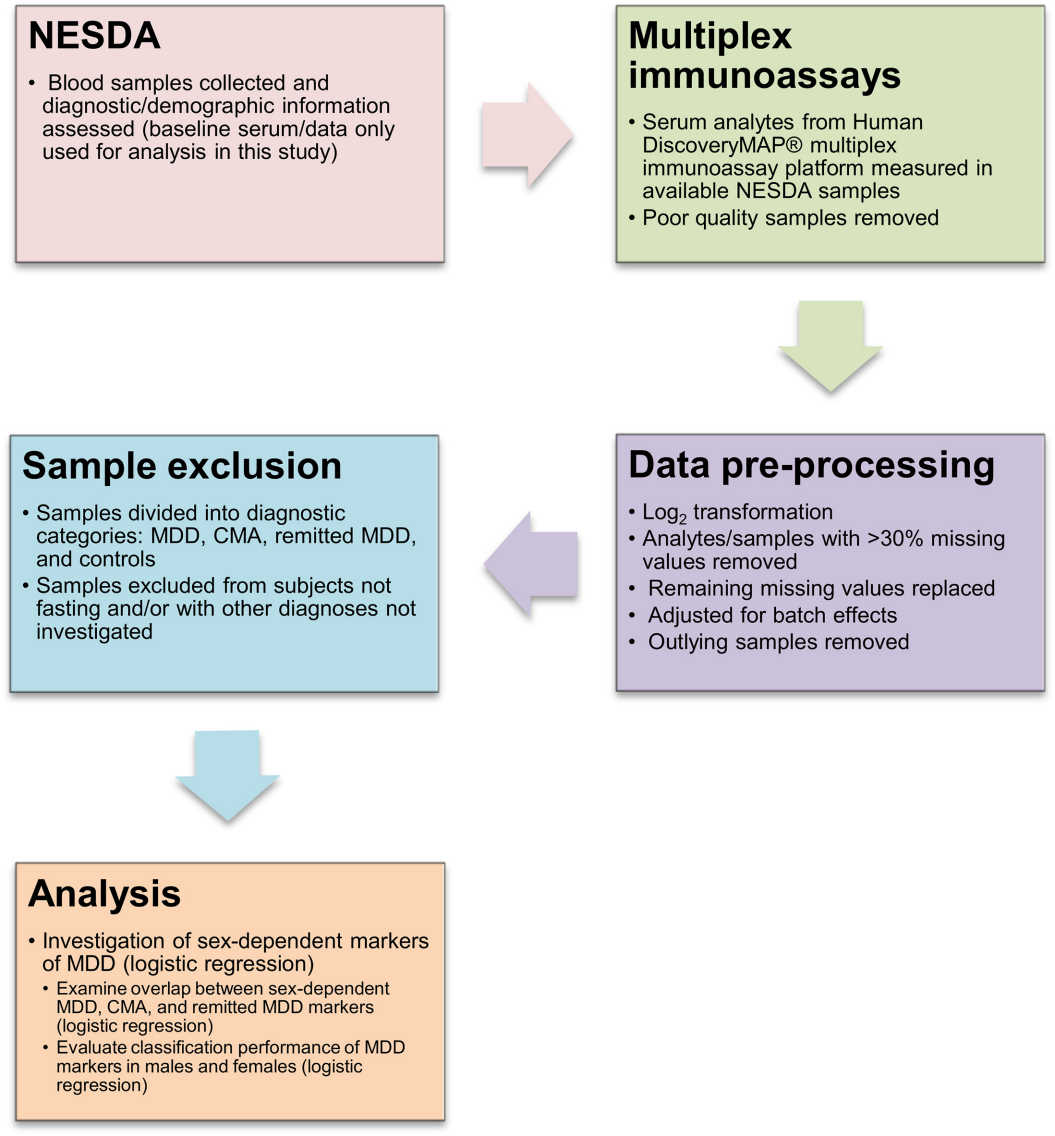
Study methods overview. A brief overview of the study methods is shown here, from sample collection to data analysis. **Abbreviations:** NESDA (Netherlands Study of Depression and Anxiety); MDD (major depressive disorder); CMA (comorbid MDD and anxiety disorder(s)).

An overview of the methods described in this section can be found in Fig 1.

### Clinical samples

Clinical samples were from the Netherlands Study of Depression and Anxiety (NESDA), in which 2,981 participants aged 18–65 years were recruited between 2004–2007 from the community (19%), general practices (54%), and mental health organizations (27%) [21] and followed up. The study protocol for NESDA was approved by the Ethical Review Board of the VU University Medical Centre and by local review boards at each participating centre (Ethical Review Boards of the Leiden University Medical Centre and the Groningen University Medical Centre). Informed written consent was given by all participants. Patients and controls were excluded when not fluent in the Dutch language and when they had a primary clinical diagnosis of other psychiatric disorders not studied in NESDA: bipolar disorder, obsessive compulsive disorder, severe substance use disorder, or psychotic disorder. In this study, serum samples and data collected from the baseline assessment were used in analyses.

Diagnoses of depressive disorders (major depressive disorder (MDD) and dysthymia) and anxiety disorders (social phobia, generalized anxiety disorder, panic disorder, and agoraphobia) were carried out during the baseline interview by specially trained research staff using the Composite Interview Diagnostic Instrument (CIDI) in accordance with Diagnostic and Statistical Manual of Mental Disorders (DSM)-IV criteria [25]. Patients with current disorders were classified as having a depressive and/or anxiety episode within the past six months. Control subjects had neither a current nor a lifetime diagnosis of the evaluated psychiatric disorders and did not develop any assessed disorder by the second year follow-up assessment. Participants completed a 30-item self-rated Inventory of Depressive Symptomatology (IDS) [26] and 21-item self-report Beck Anxiety Inventory (BAI) [27]. History of MDD and anxiety disorders and presence of first onset or recurrent MDD was determined with the CIDI. Family history of depression or anxiety in first degree relatives was assessed [28], as well as antidepressant and benzodiazepine use. These and other demographic, lifestyle, and health variables are further described in S1 Table. A summary of these variables (stratified by sex) measured during the baseline assessment is reported in S2 Table. Further details of the NESDA study design and protocol have been previously described [21].

We categorized patients into three separate groups based on diagnosis at the baseline assessment in order to reduce potential heterogeneity in sex differences between disorders. Individuals with MDD were currently experiencing an episode but were without a current comorbid anxiety disorder. Individuals with comorbid MDD and anxiety disorder(s) (CMA) had both a current MDD episode and at least one current anxiety disorder. Finally, remitted MDD patients had a lifetime diagnosis of MDD, but were not currently experiencing MDD or an anxiety disorder. Individuals diagnosed with bipolar disorder and control subjects diagnosed with MDD or anxiety disorder(s) at the second year follow-up assessment were excluded from further analysis (see also **Sample exclusion** below) in order to obtain more homogeneous patient and control groups. Diagnoses at the second year follow-up assessment were again carried out using the CIDI.

### Multiplex immunoassays

Blood samples from the NESDA baseline assessment were collected in the morning at approximately 0800 hours after an overnight fast. Only serum samples from the baseline assessment were analyzed in this study. Serum samples were stored at –80°C until analysis. Protocol for the study participants, collection and storage of clinical samples, and test methods were carried out in compliance with the Standards for Reporting of Diagnostic Accuracy (STARD) initiative. The Human DiscoveryMAP^®^ multiplex immunoassay platform (Myriad RBM; Austin, TX, USA) was used to measure the serum concentrations of 243 analytes. The Human DiscoveryMAP^®^ is a panel of pre-selected analytes designed to investigate a broad range of biological processes important in diseases, including those commonly associated with cancer, cardiovascular disease, kidney injury, neurodegenerative disorders, and inflammation and metabolic pathways (https://rbm.myriad.com; *accessed August 2015*). S3 Table contains the list of all 243 measured analytes. The data from this panel of markers was used to evaluate changes in analyte concentrations in MDD in NESDA (Bot et al. (2015) [19]). Sex-dependent analyses carried out here complement the work of Bot et al (2015) [19], which used this data but did not examine potential sex differences in markers. The panel was used to both explore markers that have previously been associated with MDD (e.g., cortisol, C-reactive protein, and thyroid stimulating hormone) and to screen markers not yet investigated. The analytes were measured in 1,840 NESDA participants. Remaining baseline NESDA participants did not provide blood, did not participate in the second year follow-up assessment, or had serum samples with insufficient volume or that were otherwise unusable. Of these, three samples were removed after internal quality control checks revealed poor quality. The assay procedure was carried out in a Clinical Laboratory Improvement Amendments (CLIA)-certified laboratory at Myriad-RBM (Austin, TX, USA). Samples were assigned to 26 plates and blinded to analysts using code numbers until all biochemical assays were completed. Assays were calibrated using standards, raw intensity measurements were converted to absolute protein concentrations, and performance was verified using quality control samples. All analyte concentrations were reported as ng/mL, pg/mL or international units (mIU), as appropriate. Average intra-assay variability was 5.6% (range from 2.5–15.8%) and inter-assay variability was 10.6% (range from 5.5–32.5%) [19].

### Data pre-processing

Data pre-processing and analysis was carried out using R (v3.1.2) [29]. Analyte assays with more than 30% missing values in the 1,837 samples were first removed. This resulted in exclusion of 72 assays from the panel of 243, leaving 171 for further analysis. Among the excluded assays was interleukin-6 (IL-6), for which 99.3% of values were below the limit of detection. A list of all 243 analytes and the percentage of missing values contained in each assay can be found in S3 Table. In addition, one sample was removed from the study which had more than 30% missing assays. Missing values for the remaining assays were replaced by the minimum or maximum analyte level for measurements below or above the limit of quantitation, respectively, as described previously [17,30]. Analyte values that were missing due to low sample volume were replaced by the mean concentration for that analyte. Results from this simple method of missing data imputation were compared to those generated by multiple imputation using the mice package in R [31]. For multiple imputation we used predictive mean matching and Bayesian linear regression imputation techniques with five imputed datasets. We replaced missing covariate data (physical activity: 5.0% missing, alcohol consumption: 0.8% missing, and recreational drug use: 0.5% missing) with the mean or most frequent value for continuous and categorical variables, respectively.

To adjust for batch effects caused by running samples on different plates, we used ComBat after log_2_ transforming analyte data, implemented in the sva package in R [32]. ComBat is an empirical Bayes method of adjusting for additive and multiplicative batch effects and has been used in microarray data [33]. Multivariate outliers were then assessed based on a robust measure of the Mahalanobis distance [34] calculated using the robust package in R [35], resulting in the removal of an additional four samples.

### Sample exclusion

**Fig 2:**
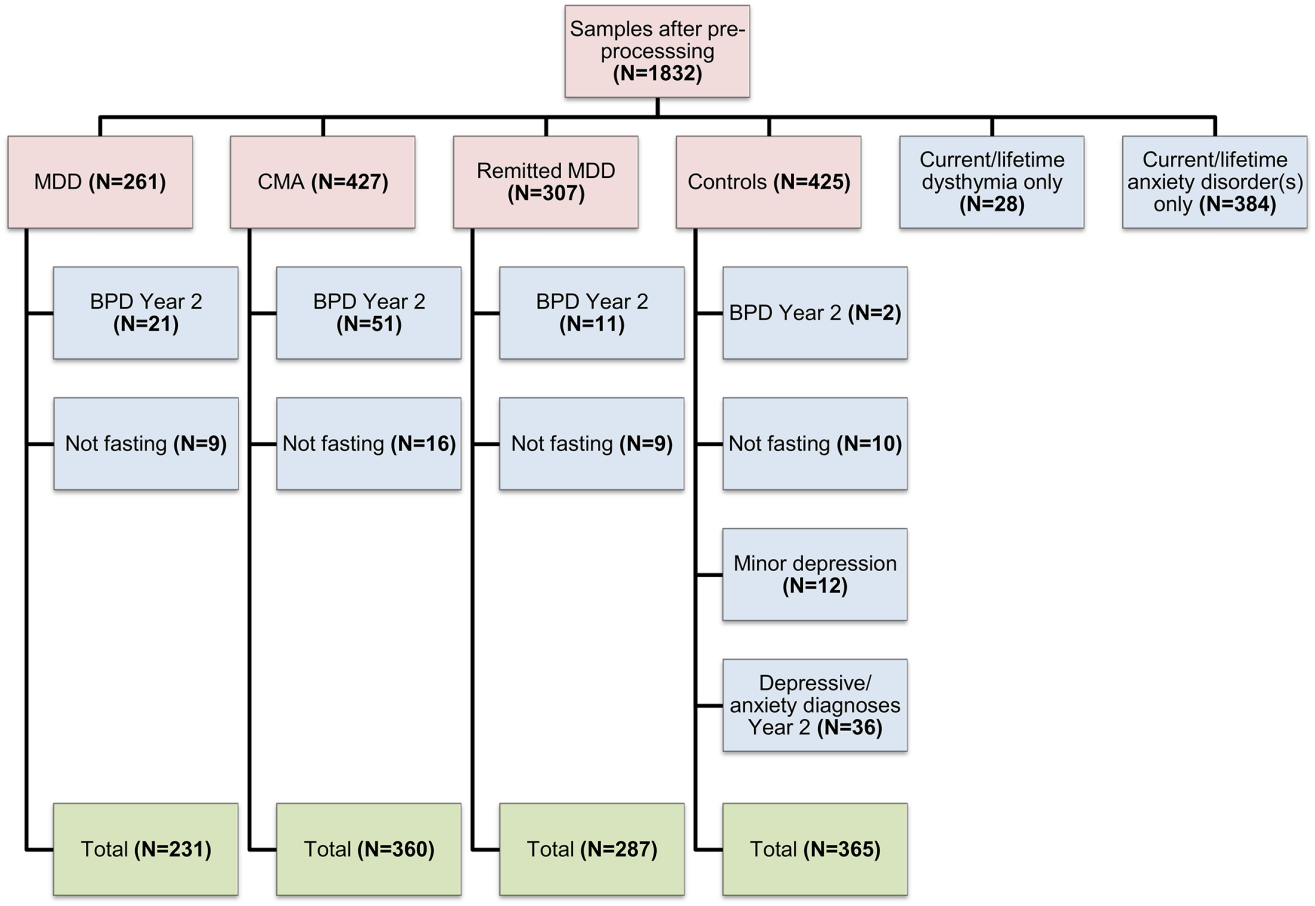
Sample exclusion for NESDA patient groups and controls after data pre-processing. Numbers are indicated in brackets. Red boxes = initial data after pre-processing; blue boxes = excluded data; green boxes = data after exclusion. Participants were excluded if they were not fasting or if they had a current/lifetime anxiety disorder or dysthymia only, current minor depression, a depressive and/or anxiety diagnosis within two years of the baseline measurement, or a lifetime bipolar disorder diagnosed at the two-year assessment. **Abbreviations:** NESDA (Netherlands Study of Depression and Anxiety); MDD (major depressive disorder); CMA (comorbid MDD and anxiety disorder(s)).

A total of 1,832 samples remained after data pre-processing. Additional samples were excluded from analysis in this study for the following reasons, as shown in Fig 2: 1) the participants had not fasted when blood was withdrawn (N = 44) or the participants were diagnosed with 2) current or lifetime dysthymia only (N = 28); 3) current or lifetime anxiety disorder(s) only (N = 384), 4) current minor depression (N = 12), 5) bipolar disorder (lifetime diagnosis) in the second year follow-up assessment (N = 85), or 6) a depressive and/or anxiety disorder episode within two years of the baseline measurement (N = 36). This left 1,243 samples (405 males and 838 females) for further analysis. These comprised control (N = 365), MDD (N = 231), CMA (N = 360), and remitted MDD (N = 287) samples.

### Data analysis

We used logistic regression with patient/control status as the outcome variable to test for sex differences in the associations between log_2_-transformed analyte concentration and log-odds of MDD diagnosis, as indicated by a significant interaction between log_2_-transformed analyte level and sex (*p<*0.05; sex-dependent markers). Other variables were also considered in analyses, including: ancestry, education, diastolic blood pressure, physical activity [36], family history of anxiety or depression [28], presence of chronic disease, use of lipid modifying agents, use of anti-inflammatory drugs, use of antihypertensive medication, collection area, BMI, age, alcohol consumption, smoking status, recreational drug use, partner status, and hormonal status (follicular phase of the menstrual cycle/luteal phase/use of OCs/postmenopausal status/other), as well as interactions between them and sex. These variables are further described in the S1 Table and summarized in S2 Table. They were selected using simultaneous forward and backward stepwise selection using Bayesian Information Criterion (BIC) selection criterion. Analytes with significant sex-analyte interaction terms (*p*<0.05; sex-dependent markers) were tested separately in males and females and classified as either male-specific, female-specific, or qualitative markers. Male-specific markers had serum concentrations that were significantly associated with log-odds of MDD in males only (*p*<0.05 in males, *p*>0.20 in females) and female-specific markers were significant in females only (*p*<0.05 in females, *p*>0.20 in males). Serum levels of qualitative markers had opposite associations with log-odds of MDD diagnosis between males and females (i.e., reduced levels of an analyte in males and increased levels of an analyte in females were associated with higher log-odds of MDD or vice versa; see S1 Appendix for a fuller description of logistic regression). Adjusted *p-*values were calculated to account for multiple testing using the Benjamini and Hochberg (1995) [37] false discovery rate (FDR) procedure and were reported for all analyses. These FDR-adjusted *p-*values are reported as *q-*values. It should be noted that FDR-based multiple testing correction procedures are less conservative than procedures to control the family-wise error rate [38]. We used the same procedure to examine sex-dependent markers of CMA and remitted MDD. The same control subjects were used as the reference population for MDD, CMA, and remitted MDD analyses. Gene ontology biological process terms for significant proteins were assessed using the UniProt website (www.uniprot.org; *accessed June 2015)* and European Bioinformatics Institute’s QuickGO online database *(*www.ebi.ac.uk/QuickGO; *accessed June 2015)*. The overlap between our serum findings, analytes with significantly different concentrations between MDD patients and controls from Bot et al. (2015) [19], and plasma analytes with significant interactions between sex and diagnosis (MDD or control) in the work of Domenici et al. (2010) [17] were also evaluated. Domenici et al. (2010) [17] measured 79 analytes in the plasma of MDD patients and controls and found 11 plasma analytes with significant sex-dependent differences in concentration between patients and controls. Plasma analytes measured in Domenici et al. (2010) are listed in S3 Table.

### Joint analyses and classification

We used a logistic regression model with forward stepwise selection using BIC [39] to define a set of analytes for classification of MDD for males and females separately. Analytes were selected from all 171 measured and retained after quality control. In order to assess the unbiased performance of the male and female models in classifying new observations, a repeated ten-fold cross-validation procedure was performed [40], followed by plotting the receiver operating characteristic (ROC) curve and measuring the area under the ROC curve (AUC). For the ten-fold cross-validation [39], the data was randomly divided into ten partitions stratified by MDD diagnosis using the sample function in the base package in R. Nine partitions were used to create a logistic regression model using forward stepwise selection with BIC of all analytes. This was then used to estimate the probability of MDD diagnosis for the observations in the last partition. This was repeated for each of the remaining partitions. The entire process of randomly dividing the data into stratified partitions, building the model using nine partitions, and evaluating in the last was done 50 times for each male and female model. This provided a better estimate of the average of all possible different splits into ten partitions [40]. Each ROC curve from the 50 repeated ten-fold cross-validations was plotted and a Wilcoxon test was used to assess whether the AUC was greater than 0.50 (performance expected by randomly guessing a class) [41]. An average ROC curve was then generated by merging all test sets into one large set [42] and the median *p-*value from Wilcoxon tests of the AUC was found. ROC curves were generated and AUCs were calculated using the ROCR package [43] in R. Wilcoxon tests were performed using the verification package in R [44].

## Results

In the NESDA cohort, females were overrepresented in the MDD (65%), CMA (72%), remitted MDD (71%), and control (62%) classifications (S2 Table). MDD, CMA, and remitted patients had significantly higher diastolic blood pressure and were more likely than controls to suffer from a chronic disease, use anti-inflammatory medication, smoke, and have a family history of depression or anxiety. There were no significant differences between males and females in MDD type (first episode or recurrent), benzodiazepine or antidepressant use, IDS or BAI scores, or presence of lifetime anxiety disorder diagnosis in MDD, CMA, or remitted MDD.

**Fig 3:**
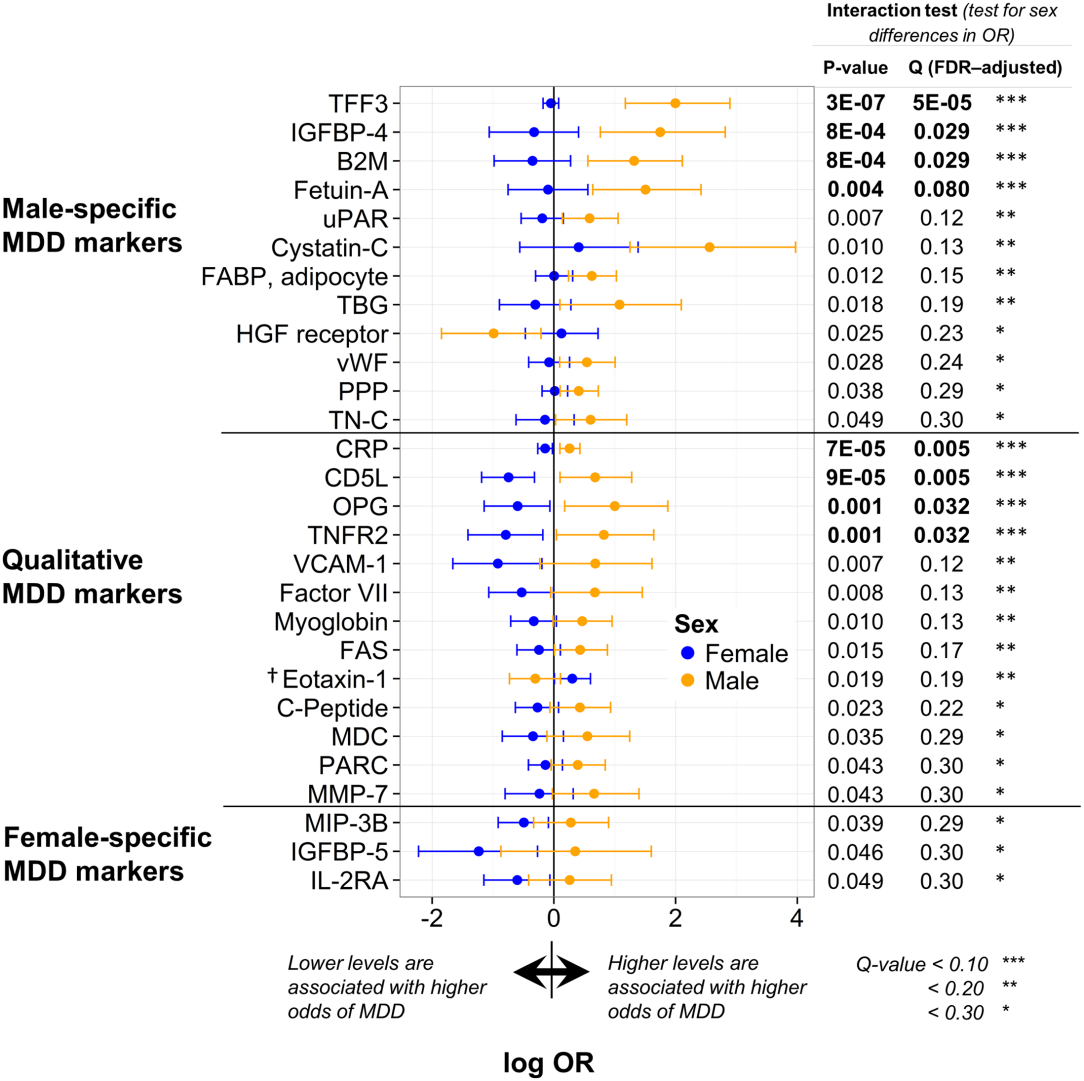
Analytes with significant interactions between log_2_-transformed serum concentration and sex in MDD compared to controls. The natural logarithm of the odds ratio (OR; the ratio of the odds of MDD diagnosis associated with a two-fold increase in the untransformed serum concentration of that analyte from the logistic model) is shown for males and females with 95% confidence intervals. *P-* and *q-*values (FDR-adjusted *p-*values) are shown for the interaction tests. **Abbreviations:** OR (odds ratio); Q (*q-*value, FDR-adjusted *p—*value); TFF3 (trefoil factor 3); IGFBP (insulin-like growth factor binding protein); B2M (β2-microglobulin); uPAR (urokinase-type plasminogen activator receptor); FABP (fatty acid-binding protein); TBG (thyroxine-binding globulin); HGF (hepatocyte growth factor); vWF (von Willebrand factor); PPP (pancreatic polypeptide); TN-C (tenascin-C); CRP (C-reactive protein); CD5L (CD5 antigen-like); OPG (osteoprotegerin); TNFR2 (tumor necrosis factor receptor 2); VCAM (vascular cell adhesion molecule); FAS (FASLG receptor); MDC (macrophage derived chemokine); PARC (pulmonary and activation-regulated chemokine); MMP (matrix metalloproteinase); MIP-3B (macrophage inflammatory protein-3β); IL-2RA (interleukin-2 receptor α). † Eotaxin-1 was no longer significant when analyzed using multiple imputation (see S4 Table).

**Fig 4:**
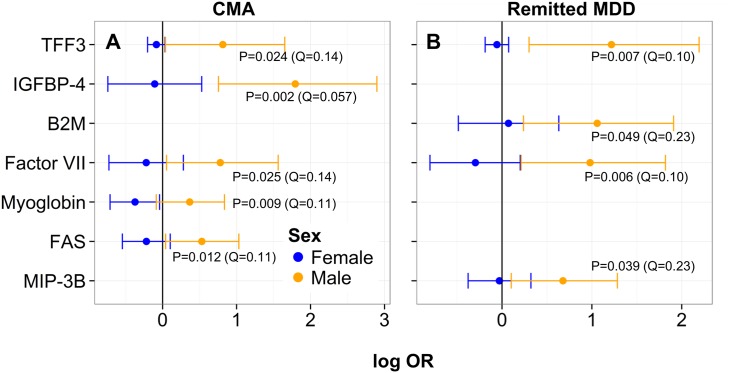
Analytes with overlapping significant interactions between sex and log_2_-transformed serum concentration in MDD and (A) CMA and (B) remitted MDD compared to controls. The natural logarithm of the odds ratio (OR; ratio of the odds of diagnosis associated with a two-fold increase in the untransformed serum concentration of that analyte from the logistic model) is shown for males and females with 95% confidence intervals. *P-* and *q-*values (FDR-adjusted *p-*values) are shown for the interaction tests. Analytes are plotted in the same order as Fig 3. **Abbreviations:** CMA (comorbid MDD and anxiety disorder(s)); OR (odds ratio); Q (*q-*value, FDR-adjusted *p—*value); TFF3 (trefoil factor 3); IGFBP (insulin-like growth factor binding protein); B2M (β2-microglobulin); FAS (FASLG receptor); MIP-3B (macrophage inflammatory protein-3β).

Twenty-eight analytes were sex-dependent markers of MDD, as defined by a significant interaction (*p<*0.05) between log_2_-transformed serum concentration and sex in the logistic regression analysis of MDD patients and controls. Results are illustrated graphically in Fig 3 and in tabular form for both simple and multiple imputation in S4 Table. After adjusting *p-*values for multiple testing, these interactions were also all significant at *q<*0.30 and eight were significant at *q<*0.10 [trefoil factor 3 (TFF3), C-reactive protein (CRP), CD5 antigen-like (CD5L), IGFBP-4, β2-microglobulin (B2M), osteoprotegerin (OPG), tumor necrosis factor receptor 2 (TNFR2), and fetuin-A in order of decreasing significance]. *Q-*values greater than 0.10 were obtained for 20 of the results, with nine additional analytes significant at *q<*0.20 [urokinase-type plasminogen activator receptor (uPAR), vascular cell adhesion molecule 1 (VCAM-1), factor VII, cystatin-C, myoglobin, fatty acid-binding protein (FABP) (adipocyte), FASLG receptor (FAS), thyroxine-binding globulin (TBG), and eotaxin-1] and the remaining 11 significant at higher *q*-values of less than 0.30 [C-peptide, hepatocyte growth factor (HGF) receptor, von Willebrand factor (vWF), macrophage derived chemokine (MDC), pancreatic polypeptide (PPP), macrophage inflammatory protein-3β (MIP-3B), pulmonary and activation-regulated chemokine (PARC), matrix metalloproteinase-7 (MMP-7), insulin-like growth factor binding protein (IGFBP)-5, tenascin-C (TN-C), and interleukin-2 receptor α (IL-2RA)]. Results were qualitatively similar to those generated using multiple imputation for analytes with missing values (S4 Table). However, it should be noted that one analyte (eotaxin-1) was no longer significant when analyzed using multiple imputation. IGFBP-5, MIP-3B, and IL-2RA were the only female-specific markers, with lower serum levels associated with increased odds of MDD in females only. IGFBP-5 participates in biological processes including cell growth, glucose metabolism, and signal transduction, while MIP-3B and IL-2RA are involved in inflammatory response and regulation of T cell proliferation. Of the remaining 25 analytes, 12 were male-specific and 13 had a qualitative interaction. With the exception of HGF receptor and eotaxin-1, higher levels of male-specific analytes and analytes with qualitative interactions were associated with increased odds of MDD in males compared to females. Many of these are involved in defence, inflammatory, and immune response [TFF3, B2M, fetuin-A, cystatin-C, FABP (adipocyte), CRP, CD5L, VCAM-1, TNFR2, FAS, eotaxin-1, C-peptide, MDC, PARC, and MMP-7]. Others, including IGFBP-4, HGF receptor, vWF, uPAR, TBG, PPP, TN-C, OPG, factor VII, and myoglobin, participate in a variety of biological processes. Among these are blood coagulation, apoptosis, signal transduction, cell adhesion, oxygen transport, regulation of cell growth, glucose metabolism, regulation of appetite, and skeletal system development. Only five analytes (TFF3, IGFBP-4, factor VII, myoglobin, and FAS) with significant interactions between sex and log_2_-transformed analyte concentration in the CMA/control and four (TFF3, B2M, factor VII, and MIP-3B) in the remitted MDD/control comparisons overlapped with the 28 significant analytes in the MDD/control comparisons (see Fig 4 and S5 Table). Two sex-dependent markers, TFF3 and factor VII, overlapped with all three conditions (MDD, CMA, and remitted MDD).

We compared these results to those of Bot et al. (2015) [19], who found 19 analytes with significantly different serum concentrations between controls and MDD patients without comorbid anxiety disorder(s) in this NESDA molecular data. Five analytes found to differ between MDD patients and controls in the work of Bot et al. (2015) [19] (cystatin-C, FABP (adipocyte), fetuin-A, IGFBP-5, and PPP) were rather found to be sex-dependent markers of MDD in this study, meaning their association with the disorder was different in males and females. Domenici et al. (2010) [17] measured nine plasma analytes that overlapped with our 28 findings in serum. One of our sex-dependent markers (MDC) also had a significant sex-diagnosis interaction (*p-*value < 0.05) in Domenici et al. (2010) [17]. Two more of our sex-dependent markers (TBG and vWF) had sex-diagnosis interaction *p-*values < 0.10 in Domenici et al. (2010) [17].

**Fig 5:**
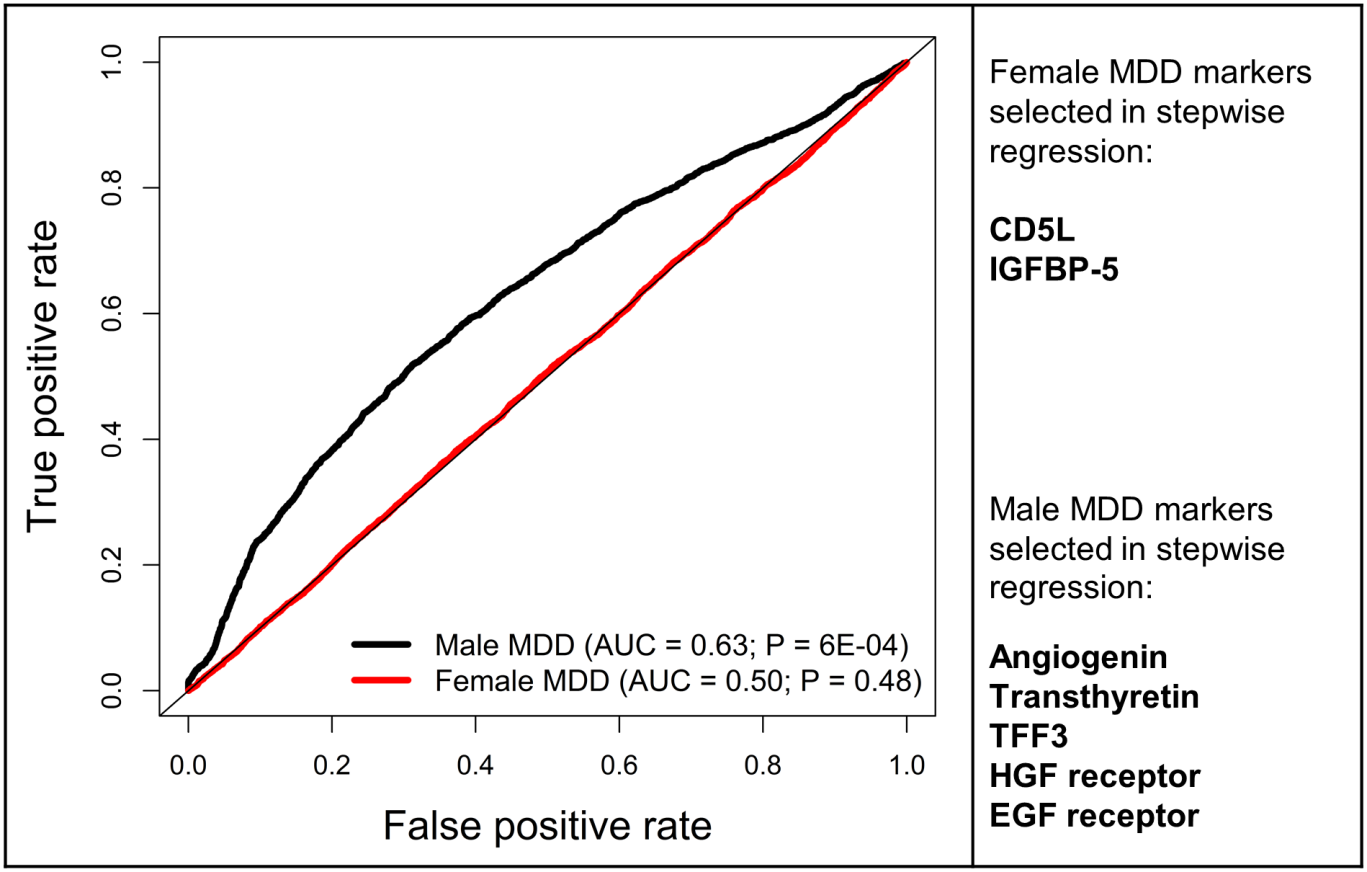
ROC curve illustrating classification of MDD patients from controls for males and females using repeated ten-fold cross validation of logistic regression with BIC forward stepwise selection of all analytes. The legend shows AUCs and median *p-*values of tests on repeated cross validations in brackets. Markers selected in forward stepwise selection of log_2_-transformed analyte data are shown in the right panel. **Abbreviations:** AUC (area under the ROC curve); P (median *p-*value); MDD (major depressive disorder); CD5L (CD5 antigen-like); IGFBP (insulin-like growth factor binding protein); TFF3 (trefoil factor 3); HGF (hepatocyte growth factor); EGF (epidermal growth factor).

Next, logistic regression models were fit for classification of current MDD compared to controls for males and females separately, using forward stepwise selection of the 171 measured analytes. Two analytes were selected for female classification (CD5L and IGFBP-5), and five were selected for male classification (TFF3, angiogenin, transthyretin, HGF receptor, and epidermal growth factor (EGF) receptor). The classification performances of the forward stepwise selection logistic regression models were then evaluated. Based on average ROC curves from the 50 repeated ten-fold cross-validations, an AUC of 0.63 was found for male classification (median *p-*value of tests 6E-04) and an AUC of 0.50 was found for female classification (median *p-*value of tests 0.48). These average ROC curves for males and females and the analytes selected using forward stepwise selection are shown in Fig 5. Plots of all 50 ROC curves from ten-fold repeated cross-validations for males and females can also be found in S1 Fig.

## Discussion

This study identified a number of sex-dependent serum markers of MDD in a large, well-characterized cohort. Previous studies have measured only a few molecules at a time testing specific hypotheses, assessed limited information about participants, and/or studied specific patient populations. NESDA provides extensive information on a number of serum analytes and sample characteristics from a large cross-section of the patient population from the community, primary care, and specialized health care settings. Importantly, it also assesses female hormonal status, comorbid disorders, and follow-up diagnoses. Previously, Bot et al. (2015) [19] showed that the serum concentrations of 19 molecules differed between MDD patients without comorbid anxiety disorder(s) and controls, including five (cystatin-C, FABP (adipocyte), fetuin-A, IGFBP-5, and PPP) found here to be sex-dependent markers. In total, we identified 28 sex-dependent markers of MDD, demonstrating that sex plays an important role in the molecular heterogeneity of MDD and that these interactions should be assessed in biomarker studies of the disorder.

The present study found strong evidence to support a link between MDD and elevated levels of certain proteins involved in immune response specifically in males, including CRP, TFF3, cystatin-C, fetuin-A, β2-microglobulin, CD5L, FAS, and TNFR2. These results had *q-*values less than 0.10, with the exception of FAS and cystatin-C. The association between elevated CRP and male MDD confirms the findings of a previous NESDA and other large cohort studies, including a meta-analysis of CRP in depression [45–49]. Other findings, however, were novel and provide further evidence of more inflammatory processes occurring in male compared to female MDD. Increased serum levels of these molecules have been found in low grade inflammation and autoimmune disease and are implicated in the function of T-cells, monocytes, and macrophages [50–57].

These findings may have consequences for hypotheses of depression proposing that inflammation causes the behavioural, neuroendocrine, and neurochemical changes in MDD [58,59]. Consistent with extensive sex differences in immune function [11], the results of this study indicate that this causal mechanism could be sex-dependent. Males may be more prone to dysregulation of acute inflammation and pro-inflammatory immune response, as suggested by the higher prevalence of males with autoimmune diseases with these characteristics [60] (despite a higher overall prevalence of autoimmune diseases in females) and greater male susceptibility to infection. Toker et al. (2005) have also suggested that stress pathways may influence inflammatory processes differently in males and females. Other lines of evidence point to parallel male-specific disruptions in inflammation in mental disorders. First-onset antipsychotic naive schizophrenia [24] and Asperger syndrome [23,61] have also been characterized by male-specific elevations in the serum concentrations of inflammatory molecules. Complex bidirectional relationships between depression and inflammation have been observed and further work will be required to elucidate the mechanisms involved.

In contrast to male findings, few female-specific MDD markers were found and these had high *q-*values. Female-specific MDD markers may have provided greater insight into the biological basis for its higher prevalence in females. There were no significant sex-dependent associations between total serum cortisol levels and MDD that would provide an indication of sex differences in HPA axis dysfunction, as found in previous studies [7–10]. There were also no sex differences in associations between MDD and thyroid stimulating hormone that may have indicated a greater prevalence of subclinical hypothyroidism in female MDD patients, as has been previously hypothesized [5]. Furthermore, female MDD classification using molecular data was no more accurate than chance (AUC = 0.50). Variability in female molecular levels may be higher or female MDD may be more biologically heterogeneous than male MDD, making biomarker discovery more difficult.

Few sex-dependent markers of CMA overlapped with sex-dependent markers of MDD, suggesting that different sex-dependent pathophysiological mechanisms may be involved in MDD and CMA. Studies should consider the presence of comorbid anxiety disorder(s) in future investigations of sex differences in MDD. Heterogeneity of anxiety disorders may contribute to these differences. Furthermore, most sex differences in MDD markers were only present during an episode and did not persist in remitted MDD. Similarly, Bot et al. (2015) [19] found changes in serum analyte levels to be more pronounced in current MDD compared to remitted MDD. Only two markers (TFF3 and factor VII) were significant sex-dependent markers in all three conditions (MDD, CMA, and remitted MDD).

Despite detecting a number of sex-dependent markers, the performance of male and female MDD classification based on serum molecular concentrations in this study was not adequate for use as a diagnostic tool. An objective biomarker test for MDD could provide earlier diagnosis and improve recognition of the disorder by physicians in primary care settings [1]. However, molecular measurements could not be used to separate female MDD patients from controls (AUC = 0.50). Male MDD patients were classified from controls with an accuracy slightly higher than chance (AUC = 0.63), raising the possibility that these could be used with other data to aid in diagnosis. These results contradicted the findings of Papakostas et al. (2013) [18] / Bilello et al. (2015) [62] and Domenici et al. (2010) [17]. Papakostas et al. (2013) [18] and Bilello et al. (2015) [62] used a panel of nine serum markers that were also measured in our study (α1-antitrypsin, apolipoprotein CIII, brain-derived neurotrophic factor, cortisol, epidermal growth factor, myeloperoxidase, prolactin, resistin and TNFR2) to achieve a test accuracy of 91% (AUC = 0.93) in separating MDD patients from controls. Domenici et al. (2010) [17] used ten plasma markers (nine of which overlapped with those measured in our serum study) and reported approximately 80% sensitivity and 75% specificity for classifying MDD patients from controls. Clearly, more work is needed to resolve these discrepancies and establish an accurate, reproducible biomarker signature for male and female MDD.

Certain limitations and future work should be considered. First, more studies are needed to validate these results given the high *q-*values obtained for many of the findings. Next, more work is needed to determine mechanisms by which these findings arise and investigate a broader range of biological pathways than is presently covered in the multiplex immunoassay panel. Although a number of inflammatory markers were evaluated, IL-6 could not be investigated here since most measurements were below the limit of detection. IL-6 is an important, well-researcher marker of inflammation that has shown a positive association with depression [49] and should be examined in future studies. Most of the sex-dependent markers of MDD in this work did not overlap with analytes found to have significant sex-diagnosis interactions in Domenici et al. (2010) [17]. This may be due to the investigation of plasma biomarkers instead of serum, the use of different statistical analyses, or the inclusion of only recurrent patients with and without comorbid anxiety disorders in Domenici et al. (2010) [17]. Second, although the present study analyzed current CMA separately, the number of different anxiety disorders included in the study and the high comorbidity between them prevented further investigation of sex differences in markers of specific anxiety disorders comorbid with MDD. Future studies should further investigate the influence of these and other features of MDD on sex-dependent markers of depression, such as prior anxiety disorder diagnoses, chronicity of MDD, and atypical and melancholic features. This may improve the classifier performance of biomarker tests. Finally, integration of this molecular data with brain imaging, genetic, and other relevant data would enable investigators to consider a greater number of factors thought to influence the higher prevalence of female MDD.

This study presents a step forward in understanding sex differences in MDD and has important implications for future studies. We found distinct differences between males and females in serum molecular markers of MDD, including male-specific associations between MDD and elevated levels of immune molecules. These findings may have consequences for inflammatory hypotheses of depression in males and females. Males may be more prone to an inflammatory basis of depression than females or inflammatory mechanisms in depression may differ between the sexes. Despite finding a number sex-dependent markers the classification accuracy of MDD using the measured molecules was low and only male MDD patients were classified with an accuracy higher than chance. Future studies of MDD will need to resolve discrepancies between the accuracies of biomarker tests produced from different studies. Robust tests may need to use different biomarkers for males and females. Finally, most sex-dependent serum molecular associations were not consistent across MDD, CMA, and remitted MDD. Sex differences in MDD markers may be state-dependent and presence of comorbid anxiety disorder(s) should be taken into account in future biomarker studies.

## Supporting Information

S1 Appendix Further description of logistic regression model. (PDF)

S1 Fig ROC curves from the 50 repeated ten-fold cross-validations illustrating classification of MDD patients from controls for males (A) and females (B). Bold, dotted lines are average ROC curves from Fig 5 in the main text.
(TIF)

S1 Table Description of variables. It should be noted that medication use was evaluated by self-reporting and inspection of drug containers used in the past month. **Abbreviations:** ATC (Anatomical Therapeutic Chemical).
(PDF)

S2 Table Female (A) and male (B) demographic, health, and lifestyle characteristics for MDD, CMA, and remitted MDD patients and controls. Values are shown as mean ± the standard deviation. Differences between controls and conditions were assessed using Welch's *t*-test (continuous data) or Fisher's exact test (categorical data). Variables in bold were significantly different (*p*<0.05) from controls. Abbreviations: MDD (major depressive disorder); CMA (comorbid MDD and anxiety disorder(s)); BMI (body mass index); MET (metabolic equivalent); OC (oral contraceptive); TCA (tricyclic antidepressant); SSRI (selective serotonin reuptake inhibitor).
(PDF)

S3 Table List of serum molecules measured with multiplex immunoassay. Analytes analyzed in this study (i.e., with <30% missing assay values) are marked in a ✓. Analytes measured in plasma in the work of Domenici et al. (2010) are marked with a ✓ in the last column.
(PDF)

S4 Table Analytes with significant interactions between log_2_-transformed serum concentration and sex in MDD compared to controls (A) and a comparison with results using multiple imputation (B). In **(A)**, a simple missing value imputation method was used (see main text). In **(B)**, interaction *p-*values from multiple imputation are also presented for assays with missing values using predictive mean matching and Bayesian linear regression imputation techniques (see also main text). Odds ratios (OR) represent the ratio of odds of MDD diagnosis associated with a two-fold increase in the untransformed serum concentration of that analyte from the logistic model. Ratio (R) represents the ratio between the geometric means of patient and control analyte concentrations. Orange boxes = male-specific analytes; white boxes = analytes with a qualitative interaction; blue boxes = female-specific analytes. **Abbreviations:** R (ratio, patient/control); OR (odds ratio); P (*p-*value); Q (*q-*value); TFF3 (trefoil factor 3); IGFBP (insulin-like growth factor binding protein); B2M (β2-microglobulin); uPAR (urokinase-type plasminogen activator receptor); FABP (fatty acid-binding protein); TBG (thyroxine-binding globulin); HGF (hepatocyte growth factor); vWF (von Willebrand factor); PPP (pancreatic polypeptide); TN-C (tenascin-C); CRP (C-reactive protein); CD5L (CD5 antigen-like); OPG (osteoprotegerin); TNFR2 (tumor necrosis factor receptor 2); VCAM (vascular cell adhesion molecule); FAS (FASLG receptor); MDC (macrophage derived chemokine); PARC (pulmonary and activation-regulated chemokine); MMP (matrix metalloproteinase); MIP-3B (macrophage inflammatory protein-3β); IL-2RA (interleukin-2 receptor α).
(PDF)

S5 Table Analytes with overlapping significant interactions between sex and log_2_-transformed serum concentration in MDD and (A) CMA and (B) remitted MDD compared to controls. Odds ratios (OR) represent the ratio of odds of the condition associated with a two-fold increase in the untransformed serum concentration of that analyte from the logistic model. Ratio (R) represents the ratio between the geometric means of patient and control analyte concentrations. **Abbreviations:** R (ratio, patient/control); OR (odds ratio); P (***p-***value); Q (***q-***value); MDD (major depressive disorder); CMA (comorbid MDD and anxiety disorder(s)); IGFBP (insulin-like growth factor binding protein); FAS (FASLG receptor); TFF3 (trefoil factor 3); MIP-3B (macrophage inflammatory protein-3β); B2M (β2-microglobulin).
(PDF)
