# Supplementary material for: Sex Differences in Serum Markers of Major Depressive Disorder in the Netherlands Study of Depression and Anxiety (NESDA)
Source: PLoS One. 2016 May 27;11(5):e0156624. doi: 10.1371/journal.pone.0156624 (PMC4883748; doi:10.1371/journal.pone.0156624)
Supplement: S1 Appendix — (PDF) [file pone.0156624.s001.pdf]

S1 Appendix. Further description of logistic regression model.

Logistic regression models the relationship between the probability of an event $[\pi(X) = \Pr(Y = 1|X)]$ and the independent variables. The logistic function is [1,2]:

$$\pi(\mathbf{x}_i) = \frac{e^{\beta_1 x_{i1} + \beta_2 x_{i2} + \beta_3 x_{i3} + \cdots + \beta_p x_{ip}}}{1 + e^{\beta_1 x_{i1} + \beta_2 x_{i2} + \beta_3 x_{i3} + \cdots + \beta_p x_{ip}}}, \qquad i = 1, \ldots, n$$

where observations are indexed with subscript i; n is the number of observations; and $\beta_1, \ldots, \beta_p$ are regression coefficients for p explanatory variables. This model can be manipulated to yield a function that is linear in the independent variables, with the left-hand side termed the logit:

$$\log\left(\frac{\pi(\mathbf{x}_i)}{1 - \pi(\mathbf{x}_i)}\right) = \beta_1 x_{i1} + \beta_2 x_{i2} + \beta_3 x_{i3} + \cdots + \beta_p x_{ip}, \qquad i = 1, \ldots, n$$

Parameters for this model are found using maximum likelihood estimation, a general method for estimating model parameters. Regression coefficients are interpreted as the change in log-odds associated with a one unit change in the independent variable, holding constant/adjusting for all other independent variables in the model. Exponentiating the coefficient gives the odds ratio (OR) associated with a one unit change in the independent variable. Since analytes are $\log_2$-transformed in this work, the exponentiated regression coefficient gives the odds ratio associated with a two-fold increase in the untransformed analyte concentration (again, adjusted for/holding constant other variables in the model). A likelihood ratio test was conducted to compare the fit of the full model with the reduced model [2]. Logistic regression and hypothesis testing was carried out using the glm function in the stats package of R [3].

Interactions between variables occur in regression analyses when the relationship between the dependent variable and an independent variable is modified by one or more independent variables. The model evaluated in this work was:

$$\log\left(\frac{\pi(\mathbf{x}_{ik})}{1 - \pi(\mathbf{x}_{ik})}\right) = \beta_{0k} + \beta_{1k}[sex_{\boldsymbol{i}}] + \beta_{2k}[y_{ik}] + \beta_{3k}[sex_{\boldsymbol{i}} \times y_{ik}] + \beta_{4k} x_{i4} + \cdots + \beta_{pk} x_{ip},$$
$$k = 1, \ldots, m, \qquad i = 1, \ldots, n$$

where y_{ik} is the $\log_2$-transformed concentration of analyte k for observation i; sex_i is the sex (male or female) of observation i; $sex_i \times y_{ik}$ is the interaction between these variables; $x_{i4}, \ldots, x_{ip}$ are $p - 3$ additional explanatory variable vectors selected in stepwise regression; $\beta_{0k}, \ldots, \beta_{pk}$ are regression coefficients; m is the number of analytes; p is the number of explanatory variables; and n is the number of observations. Testing the hypothesis $\beta_{3k} = 0$ assesses whether the relationship between the log-odds of MDD and the $\log_2$-transformed concentration of analyte k differs between males and females (again, adjusted for/holding constant other variables in the model). When this occurred, inference and estimation on β_{2k} was conducted for males and females separately:

$$OR_{k,male} = e^{\beta_{2k,male}} = \frac{odds\ of\ MDD\ in\ males\ when\ y_{raw,k(male)} = 2h}{odds\ of\ MDD\ in\ males\ when\ y_{raw,k(male)} = h},$$

$$OR_{k,female} = e^{\beta_{2k,female}} = \frac{odds\ of\ MDD\ in\ females\ when\ y_{raw,k(female)} = 2h}{odds\ of\ MDD\ in\ females\ when\ y_{raw,k(female)} = h}, \quad k = 1, \ldots, m$$

Interactions may occur when analyte levels are associated with log-odds of MDD in males only (male-specific) or in females only (female-specific). Qualitative or quantitative interactions may also occur, where the male and female analyte ORs are in the same

direction but the OR in one sex is greater (quantitative) or where the male and female ORs are in opposing directions (qualitative).

Results of the analyses performed and described here and in the main text can be found in **Fig 3/S4 Table** (MDD analysis) and **Fig 4/S5 Table** (overlap with CMA and remitted MDD).

Supplementary References

1. Menard S (2002) Applied Logistic Regression Analysis. 2nd ed. Thousand Oaks, CA: SAGE Publications, Inc. p.

2. Quinn GP, Keough MJ (2002) Experimental Design and Data Analysis for Biologists. Cambridge, UK: Cambridge University Press. p.

3. R Core Team (2014) R: A Language and Environment for Statistical Computing.
