# Supplementary figures and images for: Sex Differences in Serum Markers of Major Depressive Disorder in the Netherlands Study of Depression and Anxiety (NESDA)
Source: PLoS One. 2016 May 27;11(5):e0156624. doi: 10.1371/journal.pone.0156624 (PMC4883748; doi:10.1371/journal.pone.0156624)

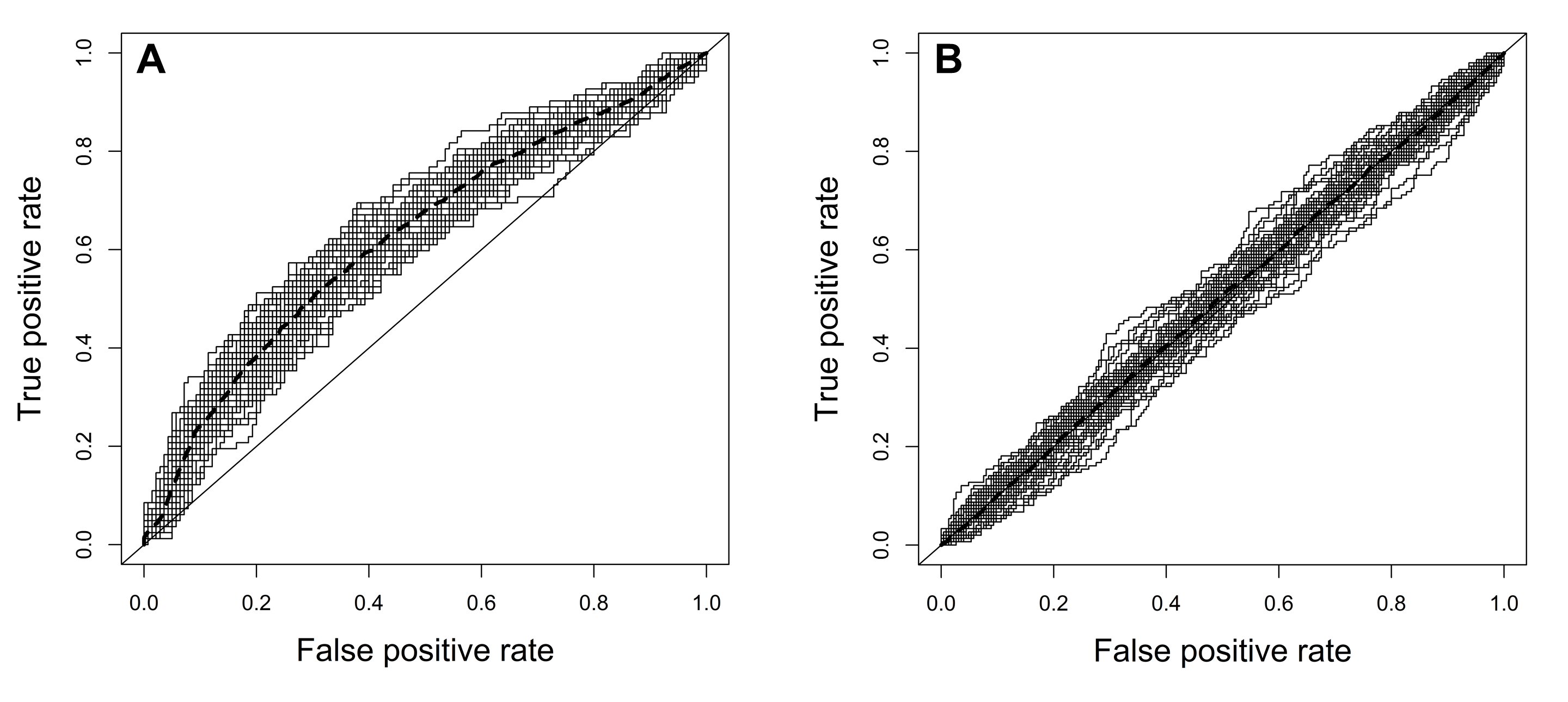

Supplement: S1 Fig — Bold, dotted lines are average ROC curves from Fig 5 in the main text. (TIF) [file pone.0156624.s002.tif]
