# Supplementary material for: Sex Differences in Serum Markers of Major Depressive Disorder in the Netherlands Study of Depression and Anxiety (NESDA)
Source: PLoS One. 2016 May 27;11(5):e0156624. doi: 10.1371/journal.pone.0156624 (PMC4883748; doi:10.1371/journal.pone.0156624)
Supplement: S1 Table — It should be noted that medication use was evaluated by self-reporting and inspection of drug containers used in the past month. Abbreviations: ATC (Anatomical Therapeutic Chemical). (PDF) [file pone.0156624.s003.pdf]

S1 Table. Description of variables. It should be noted that medication use was evaluated by self-reporting and inspection of drug containers used in the past month. **Abbreviations:** ATC (Anatomical Therapeutic Chemical)

**Variable**	**Measurement units (continuous) or categories**	**Additional information**
Collection area	Amsterdam, Leiden, or Groningen	
Recruitment method	Community, general practice, secondary mental health care	
Sex	Male, female	
Hormonal status	Male or female in the follicular phase of the menstrual cycle, luteal phase, using oral contraceptives, after menopause, and other	Assessed by self-report. Use of oral contraceptives and sex hormones (ATC code G03) was assessed by self-report. Classification of menstrual cycle phase in females [follicular (0-13 days) and luteal (14-32 days or more)] and postmenopausal status (yes/no) were also self-reported. Other female hormonal statuses included females who were hysterectomized, pregnant or breastfeeding, using sex hormones other than oral contraceptives, or with unrecorded hormonal status. These were considered as a separate classification, grouped together due to low numbers.
Age	Years	
Body mass index (BMI)	kg/m^2	
Waist circumference	Centimetres	
Ancestry	North European or other	
Education	Years	
Partner status	Yes or no	
Smoking status	Never smoked, former smoker, regular smoker, or not a regular smoker	
Alcohol consumption	Drinks/week	
Recreational drug use in the past month	Yes or no	Recreational drugs considered were cannabis, speed, cocaine, heroin, and LSD
Physical activity	Metabolic equivalent (MET)/minute	Assessed with the International Physical Activity Questionnaire
Blood pressure	mm Hg	Systolic and diastolic blood pressure measured
Presence of treated chronic somatic disease	Yes or no	Chronic diseases considered were cardiovascular disease, diabetes, lung disease, osteoarthritis, rheumatic disease, cancer, ulcer, intestinal problem, liver disease, epilepsy, thyroid gland disease and others. Presence of cardiovascular disease was assessed by self-report and confirmed by use of appropriate medication, while presence of diabetes was assessed by use of anti-diabetic medication (ATC code A10) or by fasting plasma glucose level ≥7.0 mmol l^{-1}. Other chronic diseases were treated and assessed by self-report.
Use of anti-inflammatory drugs	Yes or no	ATC codes H02, R03BA, R03AK, D07, M01A, M01B, A07EB or A07EC
Use of lipid modifying agents	Yes or no	ATC code C10
Use of antihypertensive medication	Yes or no	ATC codes C02, C03, C07, C08, or C09
Depressive symptoms	30-item self-rated Inventory of Depressive Symptomatology (IDS) score	
Anxiety symptoms	21-item self-report Beck Anxiety Inventory (BAI) score	

Family history of depressive/anxiety disorders	Yes or no	Assessed for first degree relatives using the family tree method
MDD type	First episode or recurrent	Assessed with the Composite Interview Diagnostic Instrument (CIDI)
Presence of anxiety disorder diagnosis	Lifetime or no lifetime diagnosis	Assessed with the CIDI
Use of benzodiazepines	Yes or no	ATC codes N05BA, N05CD, N03AE, and N05CF
Use of antidepressant medication	Tricyclic antidepressant (TCA), selective serotonin reuptake inhibitor (SSRI), other, mixed, or none	TCA: ATC code N06AA<br>SSRI: ATC code N06AB<br>Other: ATC codes N06AX, N06AF, and N06AG
