# Supplementary material for: Sex Differences in Serum Markers of Major Depressive Disorder in the Netherlands Study of Depression and Anxiety (NESDA)
Source: PLoS One. 2016 May 27;11(5):e0156624. doi: 10.1371/journal.pone.0156624 (PMC4883748; doi:10.1371/journal.pone.0156624)
Supplement: S2 Table — Values are shown as mean ± the standard deviation. Differences between controls and conditions were assessed using Welch's t-test (continuous data) or Fisher's exact test (categorical data). Variables in bold were significantly different (p<0.05) from controls. Abbreviations: MDD (major depressive disorder); CMA (comorbid MDD and anxiety disorder(s)); BMI (body mass index); MET (metabolic equivalent); OC (oral contraceptive); TCA (tricyclic antidepressant); SSRI (selective serotonin reuptake inhibitor). (PDF) [file pone.0156624.s004.pdf]

S2 Table. Female (A) and male (B) demographic, health, and lifestyle characteristics for MDD, CMA, and remitted MDD patients and controls. Values are shown as mean ± the standard deviation. Differences between controls and conditions were assessed using Welch's *t*-test (continuous data) or Fisher's exact test (categorical data). Variables in bold were significantly different (p<0.05) from controls. **Abbreviations**: MDD (major depressive disorder); CMA (comorbid MDD and anxiety disorder(s)); BMI (body mass index); MET (metabolic equivalent); OC (oral contraceptive); TCA (tricyclic antidepressant); SSRI (selective serotonin reuptake inhibitor).

(A)	Control (N=225)	MDD (N=149)	CMA (N=259)	Remitted MDD (N=205)
Collection sites				
Collection area % (Amsterdam/Groningen/Leiden)	18/44/37	21/38/42	19/36/46	16/54/30
Recruitment % (General population/Primary care/Specialised mental health care)	26/74/0	**11/40/50**	**7/38/55**	**38/62/0**
Lifestyle and demographic variables				
Hormonal status (Follicular/Luteal phase/OC use/ Postmenopausal/Other)	34/37/79/57/18	21/35/46/36/11	45/64/71/57/22	31/33/50/69/22
Age (years)	39±14	39.3±13	40.2±12	**43.8±13**
BMI (kg/m^2)	24.6±5	24.9±5	**26.3±6**	**25.8±5**
Waist circumference (cm)	83.3±13	84.3±12	**87.5±16**	**86±12**
North European ancestry (Yes/No)	97/3	95/5	**92/8**	99/1
Education (years)	12.8±3	12.4±3	**11.1±3**	12.5±3
Partner % (Yes/No)	78/22	**62/38**	**65/35**	74/26
Smoking % (Never/former smoker/not regular/regular smoker)	44/34/10/12	**32/30/13/25**	**29/29/13/29**	**32/36/14/19**
Alcohol % (drinks per week)	5±6	5.2±7	5.1±10	6±7
Drug use in last month % (Yes/No/NA)	4/96/0	3/97/0	5/93/2	6/94/0
Physical activity (MET/minute)	3819.4±2893	3436.6±3112	3448.4±3028	4107.2±3137
Health and medication				
Systolic blood pressure (mm Hg)	129.4±20	129.2±17	132.8±21	132.8±21
Diastolic blood pressure (mm Hg)	77.6±11	79.4±9	**81.6±13**	**80.5±11**
Chronic disease % (Yes/No)	31/69	**44/56**	**46/54**	39/61
Anti-inflammatory medication % (Yes/No)	3/97	6/94	6/94	7/93
Lipid modifying agents % (Yes/No)	3/97	1/99	5/95	6/94
Antihypertensive medication % (Yes/No)	10/90	8/92	12/88	15/85
Depression characteristics				
Symptoms (Inventory of Depressive Symptomatology; IDS)	8.6±7	**26.6±11**	**34.2±12**	**14.9±9**
Symptoms (Becks Anxiety Inventory; BAI)	4.2±5	**11.5±7**	**20.1±11**	**5.9±6**
Family history % (Yes/No)	72/28	**83/17**	**86/14**	**83/17**
MDD type % (first episode/recurrent)		43/57	43/57	54/46
Anxiety % (Lifetime/No lifetime diagnosis)		34/66	100/0	47/53
Benzodiazepine use % (Yes/No)		11/89	18/82	1/99
Antidepressant use % (TCA/SSRI/Other/Mixed/None)		1/28/9/1/61	5/32/7/1/55	1/13/1/85

(B)	Control (N=140)	MDD (N=82)	CMA (N=101)	Remitted MDD (N=82)
Collection sites				
Collection area % (Amsterdam/Groningen/Leiden)	11/51/38	13/50/37	17/39/45	15/48/38
Recruitment % (General population/Primary care/Specialised mental health care)	33/67/0	**7/35/57**	**6/33/61**	**57/43/0**
Lifestyle and demographic variables				
Age (years)	39.7±15	**44.8±12**	**43.8±11**	42.9±14
BMI (kg/m^2)	25.3±4	26.1±5	**26.5±5**	26.3±4
Waist circumference (cm)	93.1±13	96.1±14	**97.4±14**	95.8±12
North European ancestry (Yes/No)	99/1	99/1	97/3	96/4
Education (years)	12.7±3	12±3	**11.3±3**	11.8±3
Partner % (Yes/No)	74/26	76/24	71/29	78/22
Smoking % (Never/former smoker/not regular/regular smoker)	32/37/14/16	21/38/15/27	27/33/10/31	**22/28/22/28**
Alcohol (drinks per week)	10.8±11	11±15	11.6±15	10.3±12
Drug use in last month % (Yes/No/NA)	9/89/2	9/91/0	9/90/1	6/93/1
Physical activity (MET/minute)	4154.5±3551	3521.2±3118	**3291.3±2668**	4317.8±3530
Health and medication				
Systolic blood pressure (mm Hg)	144.8±18	142.5±18	143.8±26	143.9±18
Diastolic blood pressure (mm Hg)	81.7±12	83.9±10	85.2±15	83.8±11
Chronic disease % (Yes/No)	31/69	**45/55**	**54/46**	**45/55**
Anti-inflammatory medication % (Yes/No)	1/99	**9/91**	7/93	5/95
Lipid modifying agents % (Yes/No)	8/92	**20/80**	11/89	9/91
Antihypertensive medication % (Yes/No)	15/85	21/79	15/85	16/84
Depression characteristics				
Symptoms (Inventory of Depressive Symptomatology; IDS)	5.5±5	**27.6±11**	**34.3±13**	**13.8±9**
Symptoms (Becks Anxiety Inventory; BAI)	2.5±4	**11.9±9**	**20.8±12**	**4.1±5**
Family history % (Yes/No)	66/34	**82/18**	**80/20**	78/22
MDD type % (first episode/recurrent)		40/60	52/48	52/48
Anxiety % (Lifetime/No lifetime diagnosis)		22/78	100/0	46/54
Benzodiazepine use % (Yes/No)		6/94	16/84	2/98
Antidepressant use % (TCA/SSRI/Other/Mixed/None)		4/16/6/2/72	3/32/16/0/50	1/10/0/89
