# Supplementary material for: Sex Differences in Serum Markers of Major Depressive Disorder in the Netherlands Study of Depression and Anxiety (NESDA)
Source: PLoS One. 2016 May 27;11(5):e0156624. doi: 10.1371/journal.pone.0156624 (PMC4883748; doi:10.1371/journal.pone.0156624)
Supplement: S3 Table — Analytes analyzed in this study (i.e., with <30% missing assay values) are marked in a ✓. Analytes measured in plasma in the work of Domenici et al. (2010) are marked with a ✓ in the last column. (PDF) [file pone.0156624.s005.pdf]

S3 Table. List of serum molecules measured with multiplex immunoassay. Analytes analyzed in this study (i.e., with <30% missing assay values) are marked in a ✓. Analytes measured in plasma in the work of Domenici et al. (2010) are marked with a ✓ in the last column.

Analyte name	Abbreviation	UniProtKB accession/ PubChem compound identifier	% missing	Analyzed	Domenici et al. (2010)
6Ckine		O00585	0	✓	
α1-Antichymotrypsin	AACT	P01011	0	✓	
α1-Antitrypsin	AAT	P01009	0.1	✓	✓
α1-Microglobulin	A1Micro	P02760	0	✓	
α2-Macroglobulin	A2Macro	P01023	0.2	✓	✓
β2-Microglobulin	B2M	P61769	0.2	✓	✓
Adiponectin		Q15848	0.2	✓	✓
Agouti-Related Protein	AgRP	O00253	95.7		
Aldose Reductase		P15121	0.3	✓	
Alpha-Fetoprotein	AFP	P02771	32.2		✓
Amphiregulin	AR	P15514			
Angiogenin		P03950	0.2	✓	
Angiopoietin-2	ANG-2	O15123	0.1	✓	
Angiotensin-Converting Enzyme	ACE	P12821	0	✓	
Angiotensinogen		P01019	0.1	✓	
Apolipoprotein A-I	Apo A-I	P02647	0	✓	✓
Apolipoprotein A-II	Apo A-II	P02652	0	✓	
Apolipoprotein A-IV	Apo A-IV	P06727	0	✓	
Apolipoprotein B	Apo B	P04114	0	✓	
Apolipoprotein C-I	Apo C-I	P02654	0	✓	
Apolipoprotein C-III	Apo C-III	P02656	0	✓	✓
Apolipoprotein D	Apo D	P05090	7.5	✓	
Apolipoprotein E	Apo E	P02649	0.5	✓	
Apolipoprotein H	Apo H	P02749	0.2	✓	✓
Apolipoprotein(a)	Lp(a)	P08519	0.1	✓	✓
AXL Receptor Tyrosine Kinase	AXL	P30530	0	✓	
B Cell-Activating Factor	BAFF	Q9Y275	0	✓	
B Lymphocyte Chemoattractant	BLC	O43927	94.4		
Betacellulin	BTC	P35070	99.0		
Brain Derived Neurotrophic Factor	BDNF	P23560	0.1	✓	✓
Calbindin		P05937	99.3		
Cancer Antigen 125	CA 125	Q8WXI7	82.5		✓
Cancer Antigen 15-3	CA 15-3	P15941	0.1	✓	
Cancer Antigen 19-9	CA 19-9	Q9BXJ9	84.4		✓
Cancer Antigen 72-4	CA 72-4		84.4		
Carcinoembryonic Antigen	CEA	P06731	0.2	✓	✓
Cathepsin D		P07339	0.2	✓	
CD40 Antigen	CD40	Q6P2H9	0.1	✓	
CD40 Ligand	CD40L	P29965	0.4	✓	
CD5 Antigen-Like	CD5L	O43866	0.1	✓	
Cellular Fibronectin	cFib	P02751	28.7	✓	
Chemokine CC-4	HCC-4	O15467	0	✓	
Chromogranin A	CgA	P10645	2.2	✓	
Ciliary Neurotrophic Factor	CNTF	P26441	99.9		
Clusterin	CLU	P10909	0.1	✓	
Collagen IV		P02462 (a1), P08572 (a2), Q01955 (a3), P53420 (a4), P29400 (a5), Q14031 (a6)	0	✓	
Complement C3	C3	P01024	0.1	✓	✓
Complement Factor H – Related Protein 1	CFHR1	Q03591	0	✓	
Cortisol		5754	0.2	✓	
C-Peptide		P01308	0.3	✓	
C-Reactive Protein	CRP	P02741	1.3	✓	✓
Creatine Kinase-MB	CK-MB	P12277, P06732	6.1	✓	✓
Cystatin-C		P01034	0	✓	
E-Selectin		P16581	0.1	✓	
Endoglin		P17813	0	✓	
Endostatin		P39060	0.1	✓	
EN-RAGE		P80511	0	✓	
Eotaxin-1		P51671	11.6	✓	

Eotaxin-2		O00175	0.3	✓	
Eotaxin-3		Q9Y258	99.7		
Epidermal Growth Factor	EGF	P01133	0.3	✓	✓
Epidermal Growth Factor Receptor	EGFR	P00533	0.1	✓	
Epiregulin	EPR	O14944	99.0		
Epithelial Cell Adhesion Molecule	EpCam	P16422	98.6		
Epithelial-Derived Neutrophil-Activating Protein 78	ENA-78	P42830	0	✓	✓
Ezrin		P15311	92.5		
Factor VII		P08709	0.1	✓	✓
Fas Ligand	FasL	P48023	98.9		
FASLG Receptor	FAS	P25445	4.1	✓	
Fatty Acid-Binding Protein, adipocyte	FABP, adipocyte	P15090	0.1	✓	
Fatty Acid-Binding Protein, heart	FABP, heart	P05413	85.1		
Fatty Acid-Binding Protein, liver	FABP, liver	P07148	89.1		
Ferritin	FRTN	P02794, P02792	2.2	✓	✓
Fetuin-A		P02765	0	✓	
Fibrinogen		P02671, P02675, P02679	71.3		✓
Fibroblast Growth Factor 4	FGF-4	P08620	99.8		
Fibroblast Growth Factor basic	FGF-basic	P09038	99.5		✓
Fibulin-1C	Fib1C	P23142	0.4	✓	
Follicle-Stimulating Hormone	FSH	P01225, P01215	2.0	✓	
Galectin-3		P17931	0.1	✓	
Gelsolin		P06396	0.2	✓	
Glucagon		P01275	99.5		
Glucagon-Like Peptide 1, active	GLP-1 active		99.6		
Glucagon-Like Peptide 1, total	GLP-1 total	P01275	93.4		
Glucose-6-Phosphate Isomerase	G6PI	P06744	0	✓	
Glutathione S-Transferase α	GSTA	P08263	26.1	✓	
Glutathione S-Transferase Mu 1	GSTM1	P09488	95.3		
Granulocyte Colony-Stimulating Factor	G-CSF	P09919	32.0		
Granulocyte-Macrophage Colony-Stimulating Factor	GM-CSF	P04141	100		✓
Growth Hormone	GH	P01241	15.5	✓	✓
Growth-Regulated α protein	GRO-A	P09341	0	✓	
Haptoglobin		P00738	4.5	✓	
HE4		Q14508	96.0		
Heat Shock Protein 60	HSP-60	P10809	99.4		
Heparin-Binding EGF-Like Growth Factor	HB-EGF	Q99075	31.3		
Hepatocyte Growth Factor	HGF	P14210	0.3	✓	
Hepatocyte Growth Factor receptor	HGF receptor	P08581	0	✓	
Hepsin		P05981	0	✓	
Human Chorionic Gonadotropin β	hCGb	P01233	92.9		
Human Epidermal Growth Factor Receptor 2	HER-2	P04626	0	✓	
Immunoglobulin A	IgA		0.1	✓	✓
Immunoglobulin E	IgE		62.9		✓
Immunoglobulin M	IgM		0.2	✓	✓
Insulin		P01308	20.6	✓	✓
Insulin-like Growth Factor Binding Protein 1	IGFBP-1	P08833	1.4	✓	
Insulin-like Growth Factor Binding Protein 2	IGFBP-2	P18065	0	✓	
Insulin-like Growth Factor Binding Protein 3	IGFBP-3	P17936	0.1	✓	
Insulin-like Growth Factor Binding Protein 4	IGFBP-4	P22692	0.1	✓	
Insulin-like Growth Factor Binding Protein 5	IGFBP-5	P24593	0.1	✓	
Insulin-like Growth Factor Binding Protein 6	IGFBP-6	P24592	0.1	✓	
Interferon gamma	IFNγ	P01579	97.9		
Interferon-Inducible T-cell alpha chemoattractant	ITAC	O14625	30.4		
Intercellular Adhesion Molecule 1	ICAM-1	P05362	0.1	✓	✓
Interferon γ Induced Protein 10	IP-10	P02778	0.6	✓	
Interleukin-1 alpha	IL-1 alpha	P01583	30.3		✓
Interleukin-1 beta	IL-1 beta	P01584	94.2		✓
Interleukin-1 receptor antagonist	IL-1ra	P18510	1.6	✓	
Interleukin-2	IL-2	P60568	100		✓
Interleukin-2 receptor α	IL-2RA	P01589	0	✓	
Interleukin-3	IL-3	P08700	100		✓
Interleukin-4	IL-4	P05112	99.9		✓
Interleukin-5	IL-5	P05113	100		✓
Interleukin-6	IL-6	P05231	99.3		✓
Interleukin-6 receptor	IL-6r	P08887	0.1	✓	
Interleukin-6 receptor subunit β	IL-6rB	P40189	0.2	✓	
Interleukin-7	IL-7	P13232	98.5		✓
Interleukin-8	IL-8	P10145	13.1	✓	✓
Interleukin-10	IL-10	P22301	92.3		✓
Interleukin-12 Subunit p40	IL-12p40	P29460	3.5	✓	✓
Interleukin-12 Subunit p70	IL-12p70	P29459	98.6		✓
Interleukin-13	IL-13	P35225	99.3		✓

Interleukin-15	IL-15	P40933	76.9		✓
Interleukin-16	IL-16	Q14005	0.2	✓	✓
Interleukin-17	IL-17	Q16552	32.1		
Interleukin-18	IL-18	Q14116	0.7	✓	✓
Interleukin-23	IL-23	Q9NPF7	4.8	✓	
Kallikrein 5		Q9Y337	0.8	✓	
Kallikrein 7	KLK-7	P49862	99.6		
Kidney Injury Molecule-1	KIM-1	Q96D42	65.0		
Lactoylglutathione lyase	LGL	Q04760	0.1	✓	
Latency-Associated Peptide of Transforming Growth Factor β 1	LAP TGF-b1	P01137	0	✓	
Lectin-Like Oxidized LDL Receptor 1	LOX-1	P78380	28.7	✓	
Leptin		P41159	0.2	✓	✓
Luteinizing Hormone	LH	P01229, P01215	28.4	✓	
Macrophage Colony-Stimulating Factor 1	M-CSF	P09603	3.8	✓	
Macrophage Derived Chemokine	MDC	O00626	0	✓	✓
Macrophage Inflammatory Protein-1 α	MIP-1A	P10147	90.6		✓
Macrophage Inflammatory Protein-3 α	MIP-3A	P78556	81.9		
Macrophage Inflammatory Protein-1 β	MIP-1B	P13236	0.1	✓	✓
Macrophage Inflammatory Protein-3β	MIP-3B	Q99731	0	✓	
Macrophage Migration Inhibitory Factor	MIF	P14174	0.1	✓	
Macrophage Stimulating Protein	MSP	P26927	0.2	✓	
Malondialdehyde-Modified Low-Density Lipoprotein	MDA-LDL		97.5		
Maspin		P36952	99.9		
Matrix Metalloproteinase-1	MMP-1	P03956	0.9	✓	
Matrix Metalloproteinase-3	MMP-3	P08254	0.1	✓	✓
Matrix Metalloproteinase-7	MMP-7	P09237	0.2	✓	
Matrix Metalloproteinase-9		P14780	88.0		✓
Matrix Metalloproteinase-9 (total)	MMP-9	P14780	0.2	✓	
Matrix Metalloproteinase-10	MMP-10	P09238	0.2	✓	
Mesothelin	MSLN	Q13421	0.1	✓	
MHC Class I Chain-Related Protein A	MICA	Q29983	82.9		
Monocyte Chemotactic Protein 1	MCP-1	P13500	0.3	✓	✓
Monocyte Chemotactic Protein 2	MCP-2	P80075	0.4	✓	
Monocyte Chemotactic Protein 3	MCP-3	P80098	99.8		
Monocyte Chemotactic Protein 4	MCP-4	Q99616	2.0	✓	
Monokine Induced by γ Interferon	MIG	Q07325	0.3	✓	
Myeloid Progenitor Inhibitory Factor 1	MPIF-1	P55773	0.2	✓	
Myeloperoxidase	MPO	P05164	1.7	✓	
Myoglobin		P02144	0.2	✓	✓
Nerve Growth Factor beta	NGF-beta	P01138	100		
Neuron Specific Enolase	NSE	P09104	0	✓	
Neuronal Cell Adhesion Molecule	NrCAM	Q92823	2.0	✓	
Neuropilin-1		O14786	0	✓	
Neutrophil Gelatinase Associated Lipocalin	NGAL	P80188	0	✓	
N-terminal prohormone of brain natriuretic peptide	NT proBNP	P16860	6.3	✓	
Osteopontin		P10451	16.3	✓	
Osteoprotegerin	OPG	O00300	0	✓	
Pancreatic Polypeptide	PPP	P01298	0.1	✓	
Pepsinogen I	PGI	O95576	0.2	✓	
Peptide YY	PYY	P10082	70.7		
Phosphoserine Aminotransferase	PSAT	Q9Y617	0.1	✓	
Placenta Growth Factor	PLGF	P49763	82.7		
Plasminogen Activator Inhibitor 1	PAI-1	P05121	0.2	✓	✓
Platelet-Derived Growth Factor BB	PDGF-BB	P01127	0.1	✓	
Progesterone		5994	19.9	✓	
Proinsulin, intact		P01308	99.3		
Proinsulin, total		P01308	99.2		
Prolactin	PRL	P01236	0	✓	
Prostasin		Q16651	0.1	✓	
Prostate Specific Antigen, free	PSA-f	P07288	67.9		✓
Protein S100-A4	S100-A4	P26447	43.3		
Pulmonary and Activation-Regulated Chemokine	PARC	P55774	0	✓	
Receptor for advanced glycosylation end products	RAGE	Q15109	0.1	✓	
Receptor tyrosine-protein kinase erbB-3	ErbB3	P21860	0.1	✓	
Resistin		Q9HD89	0.1	✓	
S100 Calcium-Binding Protein-B	S100-B	P04271	97.9		
Serotransferrin	Transferrin	P02787	0	✓	
Serum Amyloid P-Component	SAP	P02743	0.1	✓	✓
Sex Hormone-Binding Globulin	SHBG	P04278	0.4	✓	
Sortilin		Q99523	0.1	✓	
Squamous Cell Carcinoma Antigen-1	SCCA-1	P29508	70.9		
Stem Cell Factor	SCF	P21583	0.5	✓	✓
Stromal cell derived factor 1	SDF-1	P48061	0	✓	
Superoxide Dismutase 1, soluble	SOD-1	P00441	0.8	✓	

T-Cell-Specific Protein RANTES	RANTES	P13501	0.3	✓	✓
T Lymphocyte-Secreted Protein I-309	I-309	P22362	40.3		
Tamm-Horsfall Urinary Glycoprotein	THP	P07911	0	✓	
Tenascin-C	TN-C	P24821	0.1	✓	
Testosterone (total)		6013	10.0	✓	
Tetranectin		P05452	0.2	✓	
Thrombomodulin	TM	P07204	0	✓	
Thrombospondin-1		P07996	0	✓	
Thyroglobulin	TG	P01266	25.5	✓	
Thyroid Stimulating Hormone	TSH	P01215, P01222	0.4	✓	✓
Thyroxine-Binding Globulin	TBG	P05543	0.2	✓	✓
Tissue Inhibitor of Metalloproteinases 1	TIMP-1	P01033	0.2	✓	✓
Tissue type Plasminogen activator	tPA	P00750	2.8	✓	
TNF-Related Apoptosis-Inducing Ligand Receptor 3	TRAIL-R3	O14798	0	✓	
Transforming Growth Factor alpha	TGF-alpha	P01135	96.2		
Transforming Growth Factor beta-3	TGF-beta-3	P10600	99.7		
Transthyretin	TTR	P02766	0	✓	
Trefoil Factor 3	TFF3	Q07654	0	✓	
Tumor Necrosis Factor alpha	TNF-alpha	P01375	99.6		✓
Tumor Necrosis Factor beta	TNF-beta	P01374	98.7		✓
Tumor Necrosis Factor Receptor I	TNFRI	P19438	0	✓	
Tumor Necrosis Factor Receptor 2	TNFR2	P20333	0.2	✓	✓
Tyrosine kinase with Ig and EGF homology domains 2	TIE-2	Q02763	0.1	✓	
Urokinase-type Plasminogen Activator	uPA	P00749	0.1	✓	
Urokinase-type Plasminogen Activator Receptor	uPAR	Q03405	0.6	✓	
Vascular Cell Adhesion Molecule 1	VCAM-1	P19320	0.2	✓	✓
Vascular Endothelial Growth Factor	VEGF	P15692	0.1	✓	✓
Vascular Endothelial Growth Factor B	VEGF-B	P49765	100		
Vascular Endothelial Growth Factor C	VEGF-C	P49767	0.1	✓	
Vascular Endothelial Growth Factor D	VEGF-D	O43915	92.4		
Vascular Endothelial Growth Factor Receptor 1	VEGFR-1	P17948	99.6		
Vascular Endothelial Growth Factor Receptor 2	VEGFR-2	P35968	0	✓	
Vascular Endothelial Growth Factor Receptor 3	VEGFR-3	P35916	1.0	✓	
Vitamin D-Binding Protein	VDBP	P02774	0.1	✓	
Vitamin K-Dependent Protein S	VKDPS	P07225	0	✓	
Vitronectin		P04004	0.1	✓	
von Willebrand Factor	vWF	P04275	1.1	✓	✓
YKL-40		P36222	0.2	✓	

Other analytes measured in Domenici et al. (2010): calcitonin, endothelin-1, eotaxin, erythropoietin, fatty acid-binding protein, glutathione S-transferase, lymphotactin, matrix metalloproteinase-2, prostatic acid phosphatase, serum glutamic oxaloacetic transaminase, tissue factor, thrombopoietin
