# Supplementary material for: Sex Differences in Serum Markers of Major Depressive Disorder in the Netherlands Study of Depression and Anxiety (NESDA)
Source: PLoS One. 2016 May 27;11(5):e0156624. doi: 10.1371/journal.pone.0156624 (PMC4883748; doi:10.1371/journal.pone.0156624)
Supplement: S4 Table — In (A), a simple missing value imputation method was used (see main text). In (B), interaction p-values from multiple imputation are also presented for assays with missing values using predictive mean matching and Bayesian linear regression imputation techniques (see also main text). Odds ratios (OR) represent the ratio of odds of MDD diagnosis associated with a two-fold increase in the untransformed serum concentration of that analyte from the logistic model. Ratio (R) represents the ratio between the geometric means of patient and control analyte concentrations. Orange boxes = male-specific analytes; white boxes = analytes with a qualitative interaction; blue boxes = female-specific analytes. Abbreviations: R (ratio, patient/control); OR (odds ratio); P (p-value); Q (q-value); TFF3 (trefoil factor 3); IGFBP (insulin-like growth factor binding protein); B2M (β2-microglobulin); uPAR (urokinase-type plasminogen activator receptor); FABP (fatty acid-binding protein); TBG (thyroxine-binding globulin); HGF (hepatocyte growth factor); vWF (von Willebrand factor); PPP (pancreatic polypeptide); TN-C (tenascin-C); CRP (C-reactive protein); CD5L (CD5 antigen-like); OPG (osteoprotegerin); TNFR2 (tumor necrosis factor receptor 2); VCAM (vascular cell adhesion molecule); FAS (FASLG receptor); MDC (macrophage derived chemokine); PARC (pulmonary and activation-regulated chemokine); MMP (matrix metalloproteinase); MIP-3B (macrophage inflammatory protein-3β); IL-2RA (interleukin-2 receptor α). (PDF) [file pone.0156624.s006.pdf]

S4 Table. Analytes with significant interactions between log₂-transformed serum concentration and sex in MDD compared to controls (A) and a comparison with results using multiple imputation (B). In **(A)**, a simple missing value imputation method was used (see main text). In **(B)**, interaction *p*-values from multiple imputation are also presented for assays with missing values using predictive mean matching and Bayesian linear regression imputation techniques (see also main text). Odds ratios (OR) represent the ratio of odds of MDD diagnosis associated with a two-fold increase in the untransformed serum concentration of that analyte from the logistic model. Ratio (R) represents the ratio between the geometric means of patient and control analyte concentrations. Orange boxes = male-specific analytes; white boxes = analytes with a qualitative interaction; blue boxes = female-specific analytes. **Abbreviations:** R (ratio, patient/control); OR (odds ratio); P (*p*-value); Q (*q*-value); TFF3 (trefoil factor 3); IGFBP (insulin-like growth factor binding protein); B2M (β2-microglobulin); uPAR (urokinase-type plasminogen activator receptor); FABP (fatty acid-binding protein); TBG (thyroxine-binding globulin); HGF (hepatocyte growth factor); vWF (von Willebrand factor); PPP (pancreatic polypeptide); TN-C (tenascin-C); CRP (C-reactive protein); CD5L (CD5 antigen-like); OPG (osteoprotegerin); TNFR2 (tumor necrosis factor receptor 2); VCAM (vascular cell adhesion molecule); FAS (FASLG receptor); MDC (macrophage derived chemokine); PARC (pulmonary and activation-regulated chemokine); MMP (matrix metalloproteinase); MIP-3B (macrophage inflammatory protein-3β); IL-2RA (interleukin-2 receptor α).

**(A)**	**Interaction**		**Females**				**Males**			
**Analytes**	**P**	**Q**	**OR**	**P**	**Q**	**R**	**OR**	**P**	**Q**	**R**
TFF3	3E-07	5E-05	0.95	0.46	0.52	0.90	7.36	5E-06	3E-04	1.21
IGFBP-4	8E-04	0.029	0.72	0.38	0.48	0.98	5.74	8E-04	0.008	1.12
B2M	8E-04	0.029	0.70	0.27	0.38	0.98	3.74	8E-04	0.008	1.14
Fetuin-A	0.004	0.080	0.91	0.77	0.80	0.99	4.50	9E-04	0.008	1.10
uPAR	0.007	0.12	0.83	0.28	0.38	1.00	1.80	0.012	0.058	1.20
Cystatin-C	0.010	0.13	1.50	0.41	0.49	1.02	12.91	2E-04	0.006	1.09
FABP, adipocyte	0.012	0.15	1.00	0.98	0.98	1.03	1.87	0.002	0.014	1.26
TBG	0.018	0.19	0.74	0.31	0.41	0.97	2.94	0.033	0.085	1.06
HGF receptor	0.025	0.23	1.13	0.68	0.72	1.00	0.37	0.018	0.063	0.90
vWF	0.028	0.24	0.92	0.65	0.69	1.00	1.72	0.019	0.063	1.15
PPP	0.038	0.29	1.02	0.88	0.90	1.05	1.51	0.010	0.058	1.30
TN-C	0.049	0.30	0.86	0.55	0.62	0.99	1.83	0.042	0.10	1.07
CRP	7E-05	0.005	0.87	0.019	0.063	0.73	1.29	0.002	0.014	1.76
CD5L	9E-05	0.005	0.47	8E-04	0.008	0.89	1.97	0.024	0.069	1.13
OPG	0.001	0.032	0.55	0.030	0.080	0.95	2.72	0.020	0.063	1.09
TNFR2	0.001	0.032	0.45	0.012	0.058	0.95	2.27	0.043	0.10	1.09
VCAM-1	0.007	0.12	0.40	0.013	0.058	0.96	1.97	0.15	0.23	1.05
Factor VII	0.008	0.13	0.59	0.050	0.10	0.95	1.96	0.078	0.14	1.07
Myoglobin	0.010	0.13	0.72	0.084	0.15	0.94	1.59	0.059	0.12	1.09
FAS	0.015	0.17	0.78	0.18	0.26	0.97	1.54	0.050	0.10	1.19
Eotaxin-1	0.019	0.19	1.35	0.044	0.10	1.13	0.74	0.15	0.23	0.97
C-Peptide	0.023	0.22	0.76	0.13	0.21	0.95	1.53	0.091	0.15	1.11
MDC	0.035	0.29	0.71	0.18	0.26	0.97	1.74	0.11	0.18	1.04
PARC	0.043	0.30	0.87	0.33	0.43	0.95	1.48	0.082	0.15	1.13
MMP-7	0.043	0.30	0.79	0.40	0.49	0.98	1.94	0.067	0.13	1.10
MIP-3B	0.039	0.29	0.61	0.019	0.063	0.92	1.32	0.37	0.47	1.03
IGFBP-5	0.046	0.30	0.29	0.013	0.058	0.96	1.42	0.58	0.63	1.00
IL-2RA	0.049	0.30	0.55	0.029	0.080	0.94	1.30	0.45	0.52	1.03

**(B)**	**% missing assay values** *(from **S3 Table**)*	**Simple imputation** *(from **S4 Table (A)** above)*	**Multiple imputation**	
**Analyte**			Predictive mean matching imputation	Bayesian linear regression imputation
		**Interaction *p*-value**		
uPAR	0.6	0.007	0.011	0.012
FABP, adipocyte	0.1	0.012	0.010	0.010
vWF	1.1	0.028	0.028	0.029
CRP	1.3	7E-05	5E-05	6E-05
CD5L	0.1	9E-05	2E-04	2E-04
Factor VII	0.1	0.008	0.009	0.009
FAS	4.1	0.015	0.017	0.026
C-Peptide	0.3	0.023	0.027	0.028
Eotaxin-1	11.6	0.019	0.075	0.068
MMP-7	0.2	0.043	0.041	0.039
