# Supplementary material for: Sex Differences in Serum Markers of Major Depressive Disorder in the Netherlands Study of Depression and Anxiety (NESDA)
Source: PLoS One. 2016 May 27;11(5):e0156624. doi: 10.1371/journal.pone.0156624 (PMC4883748; doi:10.1371/journal.pone.0156624)
Supplement: S5 Table — Odds ratios (OR) represent the ratio of odds of the condition associated with a two-fold increase in the untransformed serum concentration of that analyte from the logistic model. Ratio (R) represents the ratio between the geometric means of patient and control analyte concentrations. Abbreviations: R (ratio, patient/control); OR (odds ratio); P (p-value); Q (q-value); MDD (major depressive disorder); CMA (comorbid MDD and anxiety disorder(s)); IGFBP (insulin-like growth factor binding protein); FAS (FASLG receptor); TFF3 (trefoil factor 3); MIP-3B (macrophage inflammatory protein-3β); B2M (β2-microglobulin). (PDF) [file pone.0156624.s007.pdf]

S5 Table. Analytes with overlapping significant interactions between sex and log_2-transformed serum concentration in MDD and (A) CMA and (B) remitted MDD compared to controls. Odds ratios (OR) represent the ratio of odds of the condition associated with a two-fold increase in the untransformed serum concentration of that analyte from the logistic model. Ratio (R) represents the ratio between the geometric means of patient and control analyte concentrations. **Abbreviations:** R (ratio, patient/control); OR (odds ratio); P (*p*-value); Q (*q*-value); MDD (major depressive disorder); CMA (comorbid MDD and anxiety disorder(s)); IGFBP (insulin-like growth factor binding protein); FAS (FASLG receptor); TFF3 (trefoil factor 3); MIP-3B (macrophage inflammatory protein-3β); B2M (β2-microglobulin).

**(A)**	**CMA**									
	**Interaction**		**Females**				**Males**			
**Analytes**	**P**	**Q**	**OR**	**P**	**Q**	**R**	**OR**	**P**	**Q**	**R**
TFF3	0.024	0.14	0.92	0.17	0.23	0.80	2.26	0.047	0.095	1.10
IGFBP-4	0.002	0.057	0.90	0.74	0.74	1.01	6.01	0.001	0.010	1.09
Factor VII	0.025	0.14	0.80	0.39	0.43	1.00	2.18	0.041	0.095	1.10
Myoglobin	0.009	0.11	0.69	0.029	0.095	0.97	1.44	0.12	0.19	1.10
FAS	0.012	0.11	0.80	0.19	0.23	1.01	1.70	0.034	0.095	1.15
**(B)**	**Remitted MDD**									
TFF3	0.007	0.10	0.95	0.40	0.53	0.78	3.38	0.011	0.043	1.11
B2M	0.049	0.23	1.08	0.80	0.87	1.03	2.89	0.013	0.043	1.11
Factor VII	0.006	0.10	0.74	0.25	0.40	0.97	2.67	0.016	0.043	1.12
MIP-3B	0.039	0.23	0.97	0.87	0.87	1.01	1.97	0.024	0.047	1.11
